# Serum C-reactive protein level in COPD patients stratified according to GOLD 2011 grading classification

**DOI:** 10.12669/pjms.326.10905

**Published:** 2016

**Authors:** Yi-Hua Lin, Wan-Yu Wang, Su-Xian Hu, Yong-Hong Shi

**Affiliations:** 1 Yi-Hua Lin, MD, Department of Respiratory Medicine, the First Affiliated Hospital of Xiamen University, Xiamen, Fujian, China; 2 Wan-Yu Wang, MM, Department of Respiratory Medicine, the First Affiliated Hospital of Xiamen University, Xiamen, Fujian, China; 3 Su-Xian Hu, MM, Department of Respiratory Medicine, the First Affiliated Hospital of Xiamen University, Xiamen, Fujian, China; 4 Prof. Yong-Hong Shi, Department of Respiratory Medicine, the First Affiliated Hospital of Xiamen University, Xiamen, Fujian, China

**Keywords:** Chronic obstructive pulmonary disease, C-reactive protein, GOLD 2011 Grading Classification, Systemic inflammation

## Abstract

**Background and Objective:**

The Global Initiative for Chronic Obstructive Lung Disease (GOLD) 2011 grading classification has been used to evaluate the severity of patients with chronic obstructive pulmonary disease (COPD). However, little is known about the relationship between the systemic inflammation and this classification. We aimed to study the relationship between serum CRP and the components of the GOLD 2011 grading classification.

**Methods:**

C-reactive protein (CRP) levels were measured in 391 clinically stable COPD patients and in 50 controls from June 2, 2015 to October 31, 2015 in the First Affiliated Hospital of Xiamen University. The association between CRP levels and the components of the GOLD 2011 grading classification were assessed.

**Results:**

Correlation was found with the following variables: GOLD 2011 group (0.240), age (0.227), pack year (0.136), forced expiratory volume in one second % predicted (FEV_1_%; -0.267), forced vital capacity % predicted (-0.210), number of acute exacerbations in the past year (0.265), number of hospitalized exacerbations in the past year (0.165), British medical Research Council dyspnoea scale (0.121), COPD assessment test score (CAT, 0.233). Using multivariate analysis, FEV_1_% and CAT score manifested the strongest negative association with CRP levels.

**Conclusions:**

CRP levels differ in COPD patients among groups A-D based on GOLD 2011 grading classification. CRP levels are associated with several important clinical variables, of which FEV_1_% and CAT score manifested the strongest negative correlation.

## INTRODUCTION

Chronic obstructive pulmonary disease (COPD) is one of the major global health issues of the century. It affects about 10% of the population over the age of 40 years^1^ and it is predicted to be the third leading cause of death and disability in the world by 2020.^2^ The updated version of 2011 Global Initiative for Chronic Obstructive Lung Disease (GOLD) proposed crucial changes to the stratification of COPD patients.^3^ The new COPD assessment integrates a combined assessment of clinical symptoms, severity of airflow limitation, and future risks of exacerbation, classifying patients into groups A-D.

In the past few years, there has been a growing interest in the field of systemic inflammation in COPD. A number of studies have showed that there was a low grade systemic inflammation in patients with COPD, even in stable state, manifesting as elevated levels of acute phase proteins, circulating cytokines, and inflammatory cells.^4^ The systemic inflammation is correlated with reduced lung function,^4^ lower exercise capacity,^5^ increased risk of future acute exacerbations,^6^ increased risk of hospitalization,^7^ all-cause^6^-^8^ and COPD-related mortality.^7^ Also, the systemic inflammation is associated with increased risk of major comorbidities in COPD.^9^

However, little is known about the relationship between the systemic inflammation and new GOLD classification. Therefore, in the present study, we aimed to evaluate whether the levels of C-reactive protein (CRP) in COPD patients is associated with the GOLD 2011 grading classification. We also investigated the association between serum CRP and the components of the GOLD 2011 grading classification.

## METHODS

From June 2, 2015 to October 31, 2015, patients with a clinical diagnosis of COPD^3^ were recruited from the Outpatient Department of the First Affiliated Hospital of Xiamen University. Healthy controls were recruited from the physical examination center of the First Affiliated Hospital of Xiamen University. To eliminate the effect of smoking, all patients and controls were current or former smokers. All consecutive patients were screened for eligibility. Subjects were eligible for the study if they were: aged ≥40 years; current or former smokers (history ≥20 pack years); and had no history of asthma or any other lung diseases. Subjects with concomitant confounding diseases such as infection, heart failure, connective tissue disorders, cancer, hepatic or renal diseases were excluded. Patients with reading or communication disorders were also excluded. All patients had been clinically stable for at least three months. And none of them had taken corticosteroids (either oral or inhaled) for a minimum of 12 weeks prior to the commencement of the study.

The study protocol was in compliance with the Declaration of Helsinki and was approved by Ethic committee of the First Affiliated Hospital of Xiamen University. Written informed consent was obtained from all subjects before the study commenced. The study was registered at http://www.chictr.org.cn (ChiCTR-OPC-15006477). All procedures performed in studies involving human participants were in accordance with the ethical standards of the institutional and/or national research committee and with the 1964 Helsinki declaration and its later amendments or comparable ethical standards.

### COPD Assessment

All patients enrolled were assessed by a detailed questionnaire. The data collected included age, gender, weight, height, tobacco habit, lung function test results, number of acute exacerbations (characterised by the definition given by Burge and Wędzicha^10^) in the past year, number of hospitalized exacerbations in the past year, symptoms evaluated according to the modified British Medical Research Council (mMRC) dyspnoea scale^11^ and COPD assessment test (CAT).^12^

All subjects underwent a standard lung function test following the procedure for spirometry recommended by American Thoracic Society (ATS)/European Respiratory Society (ERS),^13^ and the forced expiratory volume in one second (FEV_1_) and forced vital capacity (FVC) were measured. Lung function in COPD was classified into four grades based on post-bronchodilator FEV_1_: GOLD1 (FEV_1_ ≥ 80% predicted), GOLD2 (50% ≤ FEV_1_ < 80% predicted), GOLD3 (30% ≤ FEV_1_ <50% predicted), GOLD4 (FEV_1_ < 30% predicted). The test results were incorporated into the questionnaire.

According to individualized assessment of symptoms and exacerbation risk (GOLD 2011)^3^, COPD patients in this study were classified into group A, B, C or D.

### Measurement of CRP

Fasting peripheral blood was collected. CRP was assessed in duplicate by immunonephelometry (Beckman IMMAGE 800, Beckman Coulter Inc, USA) with a lower detection limit of 0.2 mg/L. Values greater than 3.5 mg/L were considered as positive according to the manufacturer’s instructions. All measurements were performed after the final evaluation using kits with the same lot number to avoid measurement bias.

### Statistical analysis

Data were analyzed using IBM SPSS Statistics Version 19 for Windows (IBM, Chicago, IL, USA). Continuous variables were tested for normality by the Kolmogorov–Smirnov test/Shapiro–Wilk’s test. The results were presented as mean ± standard deviation (SD) for all variables that were normally distributed and as median with data range when not normally distributed. Differences between groups were analysed using the independent samples t test, while for comparisons of values that did not conform to the normal distribution, Mann–Whitney U test or Kruskal Wallis test were used. And intergroup comparisons for categorical variables were performed by Chi-square test. Correlations between parameters were calculated with Pearson’s or Spearman’s correlation test. Because of the non-normal distribution of CRP values, logarithmic transformation of CRP values was employed before performing a linear regression analysis. Stepwise regression analysis was used with log-transformed CRP as a dependent variable and variables that significantly correlated with CRP levels as independent variables. The threshold of significance was set at 5%.

## RESULTS

### Clinical Characteristics of Subjects

A total of 391 COPD patients and 50 healthy controls were included in the study. Their demographic and clinical characteristics, smoking pack-years, FEV_1_% predicted, CRP levels and comorbidities were summarized in Table-I. Patients and controls were similar in terms of age, gender, smoking pack-years and comorbidities. The baseline characteristics of the COPD patients were presented in Table-II. The distribution of COPD groups according to the 2011 GOLD classification was as follows: group A, 51 patients (13.0%); group B, 114 patients (29.2%); group C, 48 patients (12.3%); group D, 178 patients (45.5%). Patients of the four groups were similar in terms of gender, BMI, smoking pack-years and comorbidities.

**Table-I:** Comparison between COPD Patients and Control Group.

	COPD	Control	P
n	391	50	
Gender(M/F)	369/22	48/2	0.633
Age	61.56±10.39	60.20±9.05	0.379
BMI	21.42±2.24	22.93±2.22	0.000
Pack year	27.55±7.93	29.38±9.49	0.134
FEV_1_%	51.77±17.55	91.20±9.69	0.000
CRP (mg/L)	3.89(0.20-17.60)	2.56 (0.20-8.42)	0.000
Comorbidity			
Cardiovascular disease	30	3	0.672
Hypertension	28	4	0.830
Diabetes mellitus	25	3	0.914

COPD: chronic obstructive pulmonary disease, M: male, F: female, BMI: body mass index, FEV_1_%: forced expiratory volume in one second % predicted, CRP: C-reactive protein.

**Table-II:** Characteristics of the COPD Patients.

	Group A	Group B	Group C	Group D	P
n	51	114	48	178	
Gender(M/F)	48/3	107/7	46/2	168/10	0.97
Age	56.94±9.30	55.95±8.70	64.31±9.94	65.72±9.65	0.000
BMI	21.66±5.16	21.70±5.07	22.17±4.30	21.27±4.88	0.265
Pack year	28.27±9.00	27.25±7.80	25.92±8.15	27.97±7.63	0.373
FEV_1_%	71.39±9.91	68.99±8.68	42.46±5.65	37.63±8.25	0.000
FVC%	81.31±9.55	78.73±6.97	70.46±6.47	68.00±6.34	0.000
mMRC	0(0-1)	3(1-4)	1(0-1)	3(2-4)	0.000
CAT	7(4-9)	12(6-23)	7(5-9)	16(8-30)	0.000
Comorbidity					
CVD	3	7	4	16	0.783
Hypertension	4	8	3	13	0.991
DM	2	4	3	16	0.255
CRP	3.45(0.20-11.20)	3.57(0.20-8.87)	3.73(0.20-14.54)	4.33(0.20-17.60)	0.000

COPD: chronic obstructive pulmonary disease, M: male, F: female, BMI: body mass index, FEV_1_%: forced expiratory volume in one second % predicted, FVC%: forced vital capacity % predicted, mMRC: modified British Medical Research Council (mMRC) dyspnoea scale, CAT: COPD assessment test, CVD: cardiovascular disease, DM: Diabetes mellitus, CRP: C-reactive protein.

### Serum Levels of CRP

Serum CRP levels were higher in COPD patients than in controls (3.89 versus 2.56 mg/L, respectively, P<0.0001). CRP levels in the group A, B, C, and D of COPD patients were 3.45, 3.57, 3.73, 4.33 mg/L, respectively. CRP levels differed between groups A-D (P<0.0001). Pairwise comparison of CRP levels in COPD patients and control group were showed in Table-III.

**Table-III:** Pairwise comparison of CRP levels in COPD Patients and Control Group.

	Control	Group A	Group B	Group C	Group D
Control		0.004	0.000	0.001	0.000
Group A	0.004		0.522	0.467	0.000
Group B	0.000	0.522		0.772	0.000
Group C	0.001	0.467	0.772		0.014
Group D	0.000	0.000	0.000	0.014	

### Association of CRP Levels and Clinical Variables

The correlation coefficients of CRP levels with COPD assessment variables were showed in Table-IV. Statistical significant correlation was found with the following factors: GOLD 2011 group, age, pack year, FEV_1_% predicted, FVC% predicted, number of acute exacerbations and hospitalized exacerbations in the past year, mMRC grade, and CAT score.

**Table-IV:** Correlation between Age, BMI, Pack year, FEV1, FVC, mMRC, CAT and CRP levels in COPD patients.

	r	P
CRP		
COPD group	0.240	0.000
Age	0.227	0.000
BMI	0.055	0.276
Pack year	0.136	0.007
FEV_1_%	-0.267	0.000
FVC%	-0.210	0.000
Exacerbations/y	0.265	0.000
hospitalized exacerbations/y	0.165	0.001
mMRC	0.121	0.016
CAT	0.233	0.000
CVD	0.046	0.361
Hypertension	0.020	0.686
DM	-0.026	0.607

COPD: chronic obstructive pulmonary disease, BMI: body mass index, FEV1%: forced expiratory volume in one second % predicted, FVC%: forced vital capacity % predicted, mMRC: modified British Medical Research Council (mMRC) dyspnoea scale, CAT: COPD assessment test, CVD: cardiovascular disease, DM: Diabetes mellitus, CRP: C-reactive protein.

The result of stepwise regression analysis was presented in Table-V. FEV_1_% predicted and CAT score manifested the strongest negative association with log CRP levels.

**Table-V:** Results of multivariate linear regression analysis.

Predictive parameter	Regression coefficient				P

	Value	SEM	95%CI	Standardised		
FEV_1_%	-0.007	0.002	-0.011,-0.003	-0.187	0.000
CAT	0.020	0.006	0.008,0.032	0.166	0.001

With lnCRP as the dependent variable, and independent variable as COPD group, age, pack year, FEV1%, FVC%, exacerbations/y, hospitalized exacerbation/y, mMRC, CRP FEV_1_%: forced expiratory volume in one second % predicted, CAT: COPD assessment test, COPD: chronic obstructive pulmonary disease, FVC%: forced vital capacity % predicted, mMRC: modified British Medical Research Council (mMRC) dyspnoea scale.

## DISCUSSION

To date, there is no ideal disease-specific biomarker which can represent the systemic inflammation in COPD patients. Although serum CRP is not specific to COPD, it has been studied as a molecular biomarker in the stable state and during exacerbation extensively,^14^ and it remains one of the most commonly measured and inexpensive molecular biomarker in routine clinical practice. Moreover, the new method of serum CRP assessment using highly sensitive CRP (hs-CRP) kit can detect slight inflammation even in those patients without obvious symptoms. So we used hs-CRP to evaluate the low grade systemic inflammation in COPD patients in present study.

The main finding of this study was that CRP levels differed among groups A-D in a well-characterized cohort of COPD patients. The relationship between systemic inflammation and COPD severity has been frequently evaluated using the GOLD airflow obstruction classification.^5^,^14^,^15^ However, COPD is a multicomponent disease, and a multidimensional classification might provide further information beyond airflow obstruction and limitation. Meanwhile the GOLD 2011 group classification is so significant as the treatment of COPD is recommended based on this grouping.

Few studies have evaluated the systematic inflammation in COPD patients stratified according to GOLD 2011 grading classification. Kurt *et al*. reported that pentraxin 3, a novel inflammation biomarker and CRP level, did not differ between COPD A-D groups.^16^ But in that study, the number of patients in each group was only 8, 13, 2 and 24 respectively. So it cannot be ruled out that the result from that study may be attributed to chance, because a small sample size may not have insufficient statistical power to detect a slight association. In our research, the CRP levels of 391 patients with clinically stable COPD were evaluated, providing more valid and convincing results considering its relatively large sample size, which was also one of the prominent strengths of the present study.

The result of current study showed that there was evidence of low grade systemic inflammation in COPD patients across all four groups (Table-III). It reaffirmed the role of inflammation in the pathogenesis of COPD. Moreover the CRP values among patients in GOLD group D were significantly higher than in the other three groups (Table-III). It’s not surprising because the results of correlation analysis showed that CRP levels correlated with symptom scores, number of acute exacerbations in the past year, and airflow limitation severity (Table-IV).

The differences in the level of systemic inflammation in COPD patients across different groups, showed the heterogeneity of this disease. Future clinical trials are needed to determine a personalized and comprehensive therapeutic strategy for these patients. And further exploring of the potential mechanism may indicate possible alternative therapies for slowing the progress of the disease.

The present study duplicates the previous findings that patients with COPD have higher serum CRP concentrations than healthy controls.^4^,^5^,^14^,^17^ And the current study confirms the existing data that CRP levels increase when lung function worsens.^5^,^14^,^17^

Another novel and interesting findings of the current study is the correlation between CRP levels and CAT scores. The CAT is a simple questionnaire assessing and monitoring COPD, which contains eight items(cough, phlegm, chest tightness, breathlessness going up hills/stairs, activity limitation at home, confidence leaving home, sleep and energy levels) from dyspnea symptoms to health status.^12^,^18^ It associates with important clinical variables (FEV_1_% predicted, exacerbation frequency and mMRC grade)^19^ and strongly correlates with the St George’s Respiratory Questionnaire.^20^ The most plausible explanation for the association of CRP with CAT scores is its implication in the systemic inflammatory process in COPD, and particularly its association with physical capacity^5^,^21^,^22^ and depression symptoms.^23^

It is clear that CRP cannot replace COPD assessment. However, the above findings suggest that, in outpatient setting, CRP levels could perhaps be proposed as an indirect but objective estimate of CAT score and may assist in the management of COPD patients in stable state.

### Limitations of the Study

First, as a cross-sectional study, we failed to draw conclusions on the cause and effect of the associations. Second, the comorbidities were not strictly evaluated in advance, but depended solely on the medical history. Besides, there was no further analysis of the relationship between complications and systemic inflammation. Third, although there were statistically significant differences between CRP levels in different groups, the actual value of the differences was small and marginal. Finally, as there was no long-term follow-up of the patients, no prognostic data or intervention results could be obtained.

In summary, in stable COPD patients, CRP levels differ among groups A-D based on GOLD 2011 grading classification. CRP levels are associated with several important clinical variables which help predict the outcomes of patients. Among these, FEV_1_% predicted and CAT score manifested the strongest negative association. These findings reinforce serum CRP measurement in patients with COPD. Further follow-up cohort studies with larger samples would help determine the validity of these findings.
